# A descriptive study on clinical department managers’ cognition of the Plan-Do-Check-Act cycle and factors influencing their cognition

**DOI:** 10.1186/s12909-023-04293-2

**Published:** 2023-05-01

**Authors:** Xuemin Zhong, Xiaoxiao Wu, Xue Xie, Qian Zhou, Ronghua Xu, Jian Wang, Lanying He, Yawei He, Xiaobo Qiu

**Affiliations:** Second People’s Hospital of Chengdu, Sichuan, China

**Keywords:** Clinical department managers, Cognition, Deming cycle, Plan-Do-Check-act cycle, Survey

## Abstract

**Background:**

The mastery and application of the “Plan-Do-Check-Act” (PDCA) cycle by hospital clinical department managers are essential for hospitals to carry out total quality management and continuously improve medical quality. This study investigated the degree of cognition of the PDCA cycle by clinical department managers and the factors affecting their cognition.

**Methods:**

A self-designed questionnaire was used to evaluate the cognition of clinical department managers regarding the PDCA cycle in 11 municipal public Class III Grade A hospitals in Western China.

**Results:**

More than 25% of clinical department managers in the surveyed hospitals are unaware or partially aware of the PDCA cycle. Logistic regression analysis showed that sex (P = 0.049), education (P < 0.001), duty (P < 0.001), and tenure (P = 0.002) had a significant influence on managers’ cognition of PDCA. Participants who were female (P < 0.001), undergraduate (P < 0.001), head nurses, or deputy head nurses (P < 0.001), with a tenure of 5–10 years (P = 0.024) had a better cognition of the PDCA cycle. In the daily management of the department, the vast majority of managers do not implement the Check and Action steps. Among the trained managers, only 65.44% applied the complete PDCA cycle in daily management. Nearly a third of managers thought PDCA was a response to hospital demands; 82.83% of the managers need to receive PDCA cycle training, and half of them indicated a preference for online training.

**Conclusions:**

The cognition level of hospital clinical department managers regarding the PDCA cycle is relatively low, especially among the clinical department heads, and most of them are willing to accept PDCA cycle training.

**Supplementary Information:**

The online version contains supplementary material available at 10.1186/s12909-023-04293-2.

## Introduction

The level of medical technology has continuously improved with economic development and social progress, and the concurrent enhancement of hospital quality management has become an urgent focus. Introducing a scientific and advanced medical management model is also one of the main components of China’s medical and health system reform. The Evaluation Standards for Tertiary General Hospitals (2011 edition) of the Ministry of Health provides the legal basis for hospitals to improve quality management and evaluation [1]. It requires clinical department managers to carry out continuous quality improvement work through appropriate quality management improvement methods and quality management tools in combination with the principle of total quality management. The Plan-Do-Check-Act (PDCA) cycle, also known as the “quality loop,” is a general management model that originated in the 1920s. Walter A. Shewhart proposed the concept of “Plan-Do-See” (PDS), which was further developed by W. Edward Deming, an American quality management expert. He developed the “plan-do-see-check-act” cycle, also called the “Deming cycle” [2]. The Deming cycle was introduced to Japan and China in the early 1950s and late 1970s, respectively [2–4]. At present, it is widely used in improving medical quality and standardizing medical behavior, and its high efficiency in modern hospital quality management has been affirmed [5–9]. The traditional medical quality management model summarizes experience after the occurrence of medical events, focuses on the end quality management, involves less basic quality and link management, and lacks supervision of process management. The PDCA cycle is a routinized and standardized way of working. It is a management process characterized by a spiraling cycle with large loops and small loops, based on the principle of planning (P), execution or do (D), check (C), and action (A). The PDCA cycle continuously finds and solves problems to improve work efficiency [10, 11].

In recent years, China’s hospital quality management has made great progress. The scientific, standardized construction of “patient-centered” medical quality, and the quality of medical services have improved. Many studies have reported the application of the PDCA cycle management method in medical quality and safety, nursing quality, hospital infection control, equipment management, patient satisfaction, and so on. It is found that many hospitals have achieved good results in medical quality and safety management by applying PDCA cycle quality management methods. For example, the incidence of defects in clinical diagnosis and treatment, medical document writing, medical process management, and other aspects of hospitals are lower than before the implementation. The incidence of nursing quality defects decreased significantly, the pass rate of hand hygiene was significantly improved, and patients’ satisfaction with hospital work has been significantly improved. [5–12]. In China, our national hospital grade assessment requires quality and safety managers in all departments of the hospital to be proficient in using quality management tools and use them as an assessment target since 2011. However, due to an imperfect quality management system, the low overall quality of managers, defects in personnel and hospital culture, low mastery of quality management, and unscientific management procedures, there remains significant room for improvement in medical quality management [12, 13]. The mastery and application of the principles of the PDCA cycle by the managers of each hospital department are critical for total quality management and continuous improvement in medical quality. Therefore, this study investigated the degree of cognition and the factors affecting cognition of the PDCA cycle by clinical department managers in Western China municipal public Class III Grade A hospitals.

## Methods

### Ethical considerations

Ethical approval for this study was obtained from the Medical and Health Research Ethics Committee of the Second People’s Hospital of Chengdu, China. All methods were carried out in accordance with relevant guidelines and regulations.

### Content of survey instruments

A questionnaire was designed to investigate the cognition of hospital clinical department managers regarding the PDCA cycle. The design of the questionnaire was based on a literature review [5–11] and analysis and discussion with medical experts: two medical quality management experts, two nursing quality management experts, and one epidemiology and statistics expert (the four quality management experts have senior titles and more than 20 years of experience in quality management; the epidemiology and statistics expert is a doctor with more than 10 years of experience in statistical analysis). The principles of operability and sensitivity were considered in the questionnaire design, and a pre-survey was conducted. During the pre-survey, the questionnaire was sent to the managers of the administrative department to check whether the questions were clear and easy to understand and whether there were logical problems. Following the pre-survey results, the questionnaire was modified and improved, and the final questionnaire was finalized. The questionnaire was directly distributed to the clinical department managers of the sample hospital, and the purpose of the study was explained. The confidentiality of participants was ensured. The questionnaire contains 21 items and includes four parts: (1) Basic information, including sex, age, educational background, professional title, duty, and tenure; (2) Questions about the meaning of the PDCA cycle; (3) Clinical department managers’ self-evaluation of the application of the PDCA cycle; (4) The PDCA training situation and training willingness (see supplementary file). The Cronbach-α coefficient of the questionnaire’s reliability was 0.921, indicating that the questionnaire’s reliability was very good. Exploratory factor analysis suggested that the four parts and eight steps of the PDCA cycle were divided into two factors (Table 1).

**Table 1:** Results of questionnaire consistency check

Variable	Component matrix		The rotated component matrix	
Analyses the current conditions and identifies the existing problems	0.742	0.453	0.838	0.233
Identifies the causes of the problems	0.766	0.459	0.858	0.246
Identifies the major factors from various causes	0.784	0.449	0.863	0.267
Suggests solution and improvement plans related to the primary factors	0.829	0.201	0.713	0.469
Carries out the plan and measures the outcomes	0.851	0.183	0.715	0.497
Checks the results according to the requirements of the plan	0.833	-0.232	0.399	0.767
Summarizes experiences and consolidates achievements	0.853	-0.28	0.378	0.814
Turns problems that have not been solved or appear as new into the next cycle	0.783	-0.504	0.166	0.916

### Data collection

From June to August 2022, we used a questionnaire survey and multistage sampling method to investigate 11 Class III Grade A general hospitals in Western China. In China, there are currently two types of hospitals, public and private. According to relevant standards, public hospitals are not only divided into grades I - III, each grade is divided into three grades, A, B, and C, while private hospitals are only divided into grades I–III. There are more than 100 Class III Grade A general hospitals in the Sichuan Medical Alliance. We selected 11 hospitals from the Sichuan Provincial Medical Management Alliance by random sampling. The study participants were clinical department managers, including clinical department directors and head nurses. The clinical department included all internal medicine, surgery, and clinical medical technology departments. Each hospital has about 150–250 clinical department managers. The clinical manager list contains the managers’ names and job numbers. We selected 60 clinical managers from the clinical manager list of each hospital by random sampling.

Prior to the survey, the research investigators underwent standard training on the purpose of the survey, precautions, and completion methods. This ensured that a unified caliber was maintained to avoid data collection errors caused by misleading the respondents. The questionnaires were collected and coded. We eliminated questionnaires with missing and incorrect data to ensure the quality of the analyzed data. The principle of double entry was adopted to ensure the quality of data entry.

### Statistical analysis

After sorting and processing the survey data, they were entered into the EPIDATA database and subsequently imported into SPSS version 22 (IBM Corp., Armonk, NY, USA) for statistical analysis. A Cronbach-α test was used to analyze the reliability of the questionnaire. The validity of the questionnaire was analyzed by principal component analysis using exploratory factor analysis. SPSS 22.0 statistical software was used for descriptive, chi-square, and logistic regression analyses. Measurement data were described by mean ± standard deviation (x ± s). Count data were described in percentages (%). Binary and multinomial logistic regression was used to analyze the influencing factors of clinical department managers’ cognition (including sex, age, educational background, professional title, duty, and tenure) and attendance at PDCA training. The factors influencing clinical department managers’ self-evaluation of the application of the PDCA cycle were marked with the test level α = 0.05. Whether the respondents had ever known of the PDCA cycle was taken as the dependent variable, and the general information of the respondents was taken as independent variables (dummy variable processing was done for the general data of each subject). The criteria were set as entry criteria α = 0.05, exclusion criteria β = 0.10, and logistic regression analysis was performed. Taking whether participants participated in training as the dependent variable (1 indicated participation, 2 indicated non-participation) and basic information (sex, clinical working years, educational background, professional title, and duty) as independent variables, the criteria were set as follows: entry criteria α = 0.05, exclusion criteria β = 0.10. Logistic regression analysis was conducted.

## Results

### The response status of the questionnaire

A total of 660 questionnaires were distributed, and 632 were recovered. Excluding 32 questionnaires with incomplete information, a total of 600 valid questionnaires were collected. The effective recovery rate was 90.91%.

### Demographic characteristics of the respondents

Of the 600 respondents, 290 were men (48.33%) and 310 women (51.67%), with an average age of 44.08 ± 7.30 (range, 28–66) years; 96.5% of the clinical department managers have a bachelor’s degree or above. All their professional titles are middle-grade or above. Basic information is shown in Table 2.

**Table 2:** Basic information of respondents

Variable	N (%)
**Sex**	
Male	290(48.33%)
Female	310(51.67%)
**Age (X ± S, y)**	44.08 ± 7.30
**Academic credentials**	
College	21(3.5%)
Bachelor	363(60.50%)
Master	140(23.33%)
Doctor	76(12.67%)
**Professional title**	
Primary-grade	0
medium-grade	197(32.83%)
High-grade	403(67.17%)
**Duty**	
Director	168(28%)
Deputy director	232(38.67%)
Head nurse	100(16.67%)
Deputy head nurse	100(16.67%)
**Length of service (years)**	
1–5	247(41.17%)
5–10	104(17.33%)
>10	249(41.5%)

### Cognition of clinical department managers regarding the PDCA cycle

The survey results showed that 549 (91.4%) clinical department managers believed they were aware or partially aware of the PDCA cycle. Among these managers, only 72.8% answered that the PDCA cycle was the Deming cycle and 11.84% did not know the specific corresponding steps of the PDCA cycle. Only 168 managers correctly answered all the available tools for the PDCA cycle (Table 3). Among them, the top three most selected were the fishbone diagram (91.04%), checklist (82.82), and flow chart (74.04) (Table 3).

**Table 3:** The cognition of clinical department managers on the PDCA cycle

Variable	N (%)	
The meaning of the PDCA cycle		
Known	446(74.3)	
partially known	103(17.1)	
not known	51(8.5)	
PDCA cycle = Deming cycle		
Correct	437(72.8%)	
False	112(27.2%)	
The steps of the PDCA cycle		
Correct	484(88.16)	
False	65(11.84)	
The Quality control tools of the PDCA cycle		
1–3	63(11.48)	
4–6	183(33.33)	
7–9	135(24.59)	
all correct	168(30.60)	

### Analysis of factors affecting the cognition of clinical department managers regarding the PDCA

Logistic regression analysis showed that sex (P = 0.049), education (P < 0.001), duty (P < 0.001), and tenure (P = 0.002) had significant influences on managers’ cognition of the PDCA. Among them, female (OR, 2.513; 95%CI, 1.166 to 5.415; P = 0.019), bachelor’s degree (OR, 0.336; 95% CI, 0.138 to 0.817; P = 0.016), head nurse or deputy head nurse (OR, 8.063; 95%CI, 3.10 to 20.974; P < 0.001), and tenure of 5–10 years (OR, 3.598; 95% CI, 1.468 to 8.656; P = 0.004) had a better cognition of the PDCA cycle (Table 4).

**Table 4:** Analysis of factors affecting hospital managers’ cognition of PDCA

Variable	X2	P	Odds ratio	95% Confidence interval	
				lower	upper
Sex	6.031	0.019	2.513	1.166	5.415
Academic credentials	28.681	0.016	0.336	0.138	0.817
Professional title	0.679	0.739	1.105	0.614	1.989
Duty	48.657	0.000	8.063	3.100	20.974
Length of service (years)	16.434	0.004	3.589	1.488	8.656

### Clinical department managers’ self-evaluation of the application of the PDCA cycle

In daily management, 398 managers of clinical departments often performed the P step of the PDCA, 383 managers often performed the P and D steps, 335 managers performed the P, D, and C steps, and only 277 managers carried out the entire process of the PDCA cycle. Most managers failed to perform step A. Although 11 managers did not know the meaning of the PDCA cycle, they still implemented the complete PDCA cycle based on their experience in management; of these, 104 managers who had not been trained in PDCA regularly applied the PDCA cycle in its entirety (Table 5).

**Table 5:** Clinical department managers’ self-evaluation of the application of the PDCA cycle

Variable		N (%)		
		Always/ Almost Always	Sometimes	Never/Almost never
P1	Analyses the current conditions and identifies the existing problems	481(80.17%)	113(18.83%)	6(1%)
P2	Identifies the causes of the problems	475(79.17%)	119(19.83%)	6(1%)
P3	Identifies the major factors from various causes	481(80.17%)	112(18.67%)	7(1.16%)
P4	Suggests solution and improvement plans related to the primary factors	435(72.5%)	160(26.67%)	5(0.83%)
D	Carries out the plan and measures	444(74%)	148(24.67%)	8(1.33%)
C	Checks the results according to requirements of the plan	396(66%)	196(32.67%)	8(1.33%)
A1	Summarizes experiences and consolidates achievements	381(63.5%)	204(34%)	15(2.5%)
A2	Turns problems that have not been solved or appear newly into the next cycle	324(54%)	237(39.5%)	39(6.5%)

### Factors influencing the application of the PDCA cycle by managers of clinical departments

Logistic regression analysis showed that sex (P = 0.048), duty (P = 0.006), and tenure (P = 0.003) had a significant impact on the application of the PDCA cycle by clinical department managers. Descriptive statistical analysis showed that female (OR, 2.223; 95%CI,1.021 to 4.838; P = 0.044), head nurse or deputy head nurse (OR, 2.630; 95%CI, 1.101 to 6.283; P = 0.030), and tenure > 10 years (OR, 0.360; 95%CI, 0.159 to 0.817; P = 0.015) had a positive effect on the application of the PDCA cycle by managers of clinical departments (Table 6).

**Table 6:** Analysis of factors influencing the application of the PDCA cycle

Variable	X2	P	Odds ratio	95% Confidence interval		
				Lower	upper	
Sex	22.483	0.044	2.223	1.021	4.838	
Academic credentials	44.263	0.259	1.877	0.629	5.600	
Professional title	17.690	0.173	0.174	0.014	2.151	
duty	29.207	0.030	2.630	1.101	6.283	
Length of service (years)	49.889	0.015	0.360	0.159	0.817	

### The situation of PDCA cycle training for managers of clinical departments

Only 272 of the 600 respondents had received training in the PDCA cycle. Of the 272, 208 received training in hospitals, 48 received training only once, and only 141 managers received training more than three times. Among the trained managers, only 65.44% applied the complete PDCA cycle in daily management. Nearly a third of managers thought PDCA was a response to hospital demands, which means they think that the PDCA cycle does not affect department management, but that the hospital requires a theme to apply the PDCA cycle management (Table 7).

**Table 7:** The Situation of PDCA Cycle Training for Managers of Clinical Departments

Variable	N (%)
**Whether to participate in PDCA cycle training**	
Yes	272(45.33%)
No	328(54.67%)
**Ways to learn**	
Hospital training	208(76.47%)
other institutions	22(8.09%)
self-study	42(15.44%)
**Number of training sessions**	
1	48(17.65%)
2	56(20.59%)
3	27(9.93%)
>3	141(51.83%)
Application and evaluation of the PDCA cycle by trained managers	
Have you applied the complete PDCA cycle to your work?	
Yes	178(65.44%)
Not certain	92(33.82%)
No	2(0.74%)
Can you accurately apply the PDCA cycle?	
Yes	202(74.26%)
Not certain	70(25.74%)
No	0
Can PDCA help solve clinical problems?	
Yes	235(86.40%)
Not certain	37(13.60%)
No	0
To meet the demands of hospital work?	
Yes	162(27%)
No	438(73%)

### Factors affecting clinical department managers’ training attendance on the PDCA cycle

Logistic regression analysis showed that differences in duty and tenure had a significant impact on the PDCA cycle training attendance of clinical department managers. Statistical description analysis showed that head nurses or deputy head nurses (OR, 0.275; 95%CI, 0.153 to 0.497; P < 0.001) and managers of clinical departments with a tenure of 5–10 years (OR, 0.489; 95%CI, 0.268 to 0.890; P = 0.019) were related to participating in more PDCA cycle training (Table 8).

**Table 8:** Analysis of the factors affecting attendance at training on the PDCA cycle

Variable	X2	P	Odds ratio	95% Confidence interval	
				Lower	upper
Sex	1.313	0.507	1.317	0.584	2.972
Academic credentials	6.751	0.064	0.444	0.187	1.049
Professional title	0.750	0.270	0.560	0.199	1.571
Duty	16.908	0.000	0.275	0.153	0.497
Length of service (years)	10.210	0.019	0.489	0.268	0.890

### The willingness of clinical department managers to participate in the PDCA training cycle

A total of 497 (82.83%) of the managers are willing to learn further about the PDCA cycle and chose both online and offline training forms (Table 9).

**Table 9:** The willingness of clinical department managers to participate in PDCA cycle training

	N(%)
The willingness to participate in the training of PDCA cycle	
YES	497(82.83%)
NOT certain	76(12.67%)
No	27(4.5%)
Online or offline	
Online	296(49.33%)
Offline	277(50.67%)

## Discussion

Our investigation revealed that more than a quarter of clinical department managers were unaware or partially aware of the PDCA cycle. In the daily management model of the department, only 277 carried out the entire process of the PDCA cycle. Most managers failed to perform step A. Among the trained managers, only 65.44% applied the complete PDCA cycle in daily management. Nearly a third of managers thought the PDCA was a response to hospital demands. Finally, 82.83% of the managers were willing to receive PDCA cycle training, and half of them indicated a preference for online training.

Relevant statistics show that 75% of medical errors in China come from management system errors, and an important reason for errors is a lack of cognition of quality management tools. With the rapid development of medical levels, it is no longer possible to manage only through experience [12]. Adapting to the needs of current hospital management, the introduction of advanced quality management tools and methods has become an inevitable trend in hospital progress and development [14, 15]. According to the survey results, our hospital managers were generally unaware of medical quality management tools. More than 25% were unaware or partially aware of the PDCA cycle. Nearly a third of managers thought PDCA was a response to hospital demands. An article on the state of knowledge of quality management tools among nursing managers found that 95.7% of nursing managers knew at least one quality management tool, of which 59.1% of nursing managers knew more than three quality management tools [15]. As we found in the daily management process, nursing managers have a better understanding and application of quality management tools than department directors. Therefore, it is suggested that we should pay more attention to the management of directors of clinical departments and improve the quality of medical care by strengthening scientific management models. Furthermore, a small sample size study of 44 nurse quality managers found that 77.27% of the nurse quality managers were not familiar with quality management tools [16]. Consistent with previous studies, although some managers realize the importance of medical quality management knowledge, they are still unable to practically apply quality tools due to a lack of medical quality management knowledge [16, 17].

The factors that affect the recognition of quality management tools are qualifications, duties of administrators, office terms, and having to attend training. The more senior the manager’s title, the longer the office term, the more training received, and the higher the awareness of quality management tools. The reason may be that managers with lower professional titles, shorter tenure, and shorter training time have relatively less knowledge of medical quality management tools. Some managers attain a certain level of technical expertise in quality management but do not realize that quality management tools apply to clinical department administration and can also be used to improve the quality of clinical care [18]. In addition, due to busy work conditions, some managers are unwilling to spend time learning and understanding the quality management tools; hence, they are unable to participate in their application [16].

PDCA cycle includes eight steps. Most managers only carried out steps P and D and did not carry out steps C and A timeously. However, step C is the most critical step in the PDCA cycle and determines whether the management model can be continuously improved and moved forward. In addition, we found an interesting phenomenon. Although 11 managers in the survey did not know the meaning of the PDCA cycle, they still implemented the complete PDCA cycle based on their experience in management. There were 104 managers who frequently applied the PDCA cycle in its entirety, even though they had not undergone the relevant training. This indicates that the PDCA cycle actually runs through daily management, but that there is no systematic training on the PDCA cycle, therefore most managers cannot complete the entire PDCA cycle in their daily management. Our research subdivided each step of the PDCA cycle and carefully analyzed the step in the clinical management process that managers failed to implement, which was different from other related research [5–18].

Many studies have confirmed that using quality management tools can effectively reduce the incidence of adverse events such as falls, administration errors, and pressure ulcers [5–9]. For the most part, managers still think the PDCA cycle can assist clinical problem-solving by applying the correct, appropriate, practical, and effective quality management tools. In addition, managers also believe that the use of quality management tools to execute continuous quality improvement work can promote improved hospital management. In this way, fine management becomes a powerful force to promote hospital quality management [19, 20]. Our study found that less than 50% of the managers had received training, and only 65.44% of the managers who had received training applied the complete PDCA cycle in their daily management work. A study on nursing managers’ awareness of quality control tools found that 17.2% of nursing managers had not received training on quality control tools [15]. Another study from a hospital in Shanghai found that all surveyed hospital administrators had undergone PDCA cycle training [16]. In comparing our research, we found that the training of PDCA cycle for clinical department managers in Western China is far from sufficient, especially for the directors. Maybe government departments should arrange knowledge training on PDCA cycles as soon as possible [16, 21, 22]. Our research also found that nearly 85% of managers were willing to receive PDCA cycle training, and equally as many chose online or offline training. Therefore, hospitals can hold both online and offline activities to ensure that clinical managers are involved in the training.

## Conclusion

This study found that the cognition level of hospital clinical department managers regarding the PDCA cycle is relatively low, especially among the clinical department heads, and that most managers are willing to undergo training. Therefore, medical institutions should strengthen training, focus on the concept and usage of PDCA cycle training, and conduct the training online or offline according to the situation. Our study is novel because (1) previous studies provided clinical examples of the PDCA cycle, including the management in clinical diagnosis and treatment behavior, medical document writing, medical process management, the incidence of nursing quality defects, the pass rate of hand hygiene, and patients’ satisfaction with hospital work, while our study investigated the cognition of clinical department managers; (2) most studies involved investigations with nursing managers, whereas we surveyed all clinical department managers, including directors and head nurses, (3) Our questionnaire is not limited to the concept of the PDCA cycle. We divided the PDCA cycle into eight practical steps to allow managers of clinical departments to evaluate the gap between their management methods and scientific management methods.

Our study also has some limitations. The scope of the research was a primary investigation in Western China, which limits our results in terms of generalizability. Future research should conduct a nationwide survey in different countries. Second, the sample size of this study is not large enough. Third, we only investigated third-grade and Grade A general hospitals, Grade B or Grade II hospitals were not included in the survey. Fourth, respondents evaluated their performance and may therefore be biased. In the future, we will execute studies with more hospitals and larger sample sizes, and we can conduct intervention trials with PDCA cycle application in areas like medical quality and safety, nursing quality, hospital infection control, equipment management, patient satisfaction, and so on.

## Electronic supplementary material

Below is the link to the electronic supplementary material.

Supplementary Material 1

Supplementary Material 2

## Data Availability

The datasets used and/or analyzed during the current study are available from the corresponding author upon reasonable request.
