# Supplementary material for: A descriptive study on clinical department managers’ cognition of the Plan-Do-Check-Act cycle and factors influencing their cognition
Source: BMC Med Educ. 2023 May 1;23:294. doi: 10.1186/s12909-023-04293-2 (PMC10152598; doi:10.1186/s12909-023-04293-2)
Supplement: Supplementary file 1 — Supplementary Material 1 [file 12909_2023_4293_MOESM1_ESM.docx]

Note: This survey concerns your views on department management. The information will help you to understand the mode of daily management, only for the use of this study, the personal information involved in the survey is confidential.

questionnaire

**The basic information**

1.Your gender:

1. Male,
2. female

2.Your age:（）year

3.Your education:

A.Junior college

B.Bachelor's

C.Master's

D.Doctor's degree

4.Your position:

A.Clinical Department

B.medical technology Department

C.administrative Department

5.Your title:

A.Senior

B.Intermediate

C.junior

6.Your position:

A.Director

B.Deputy director

C.head nurse

D.deputy head nurse

7.Your working years as a management post:

A.less than 5 years

B.5-10 years

C.more than 10 years

**Awareness of PDCA cycle**

8.Do you know the concept of PDCA cycle:

1. aware
2. partially aware
3. not aware (jump to questions 12)

9.PDCA cycle is also known as () cycle:

A.Deming

B.Xiuhart

C.Ishikawa Kaoru

D.Feigenbaum

10.The specific steps of PDCA cycle are as follows:

A.Action, disposal, inspection and planning

B.Disposal, inspection, planning and action

C.Inspection, planning, action and disposal

D.Plan, act, inspect and dispose

11.PDCA cycle can be applied to the tools are: check list, fishbone chart, permutation chart, control chart, histogram, scatter chart, hierarchical method, flow chart, Gantt chart, countermeasure chart

**Clinical department managers' self- evaluation of the application of the PDCA cycle**

12.In the process of department management, have you done the following related work to solve the existing problems in the department(table 1)

Table 1 Clinical department managers' self- evaluation of the application of the PDCA cycle

|  | Always/ Almost  Always | sometimes | Never/Almost  never |
| --- | --- | --- | --- |
| Analyses the current conditions and finds out the existent problems |  |  |  |
| Identifies various causes resulting in those problems |  |  |  |
| Identifies the major factors from various causes |  |  |  |
| Works out the solution and improvement plan according to the major factors |  |  |  |
| Carries out the plan and measures |  |  |  |
| Checks the results according to requirements of the plan |  |  |  |
| Summarizes experiences and consolidates achievements |  |  |  |
| Turns problems that haven’t been solved or appear newly into the next cycle |  |  |  |

**Training on the PDCA cycle**

13.Have you learned PDCA cycle:

1. Yes
2. no (Skip to question 19)

14.How have you ever learned PDCA cycle:

1)hospital training

2)other institutions

3)self-study

15.How many times have you participated in PDCA cycle related training:

A.1 time,

B.2 times,

C.3 times,

D.3 times or more

16.Do you apply the complete PDCA cycle steps to your daily management: often, occasionally, no

17.Do you think you can accurately use PDCA cycle for quality problem analysis:

A.Yes

B.not sure

C.no

18. Do you think PDCA cycle can help you effectively solve daily management problems:

A.yes

B.not sure

C.no

19.Do you think PDCA cycle analysis is to meet the management requirements of the hospital?

1. Yes
2. no

20.Are you willing to participate in PDCA cycle training:

1. Yes
2. not sure
3. no(Skip to the end)

21.Which form of training do you think is most acceptable: A.online

B.offline
